# Human Intestinal Dendritic Cells Decrease Cytokine Release against *Salmonella* Infection in the Presence of *Lactobacillus paracasei* upon TLR Activation

**DOI:** 10.1371/journal.pone.0043197

**Published:** 2012-08-14

**Authors:** Miriam Bermudez-Brito, Sergio Muñoz-Quezada, Carolina Gomez-Llorente, Esther Matencio, María J. Bernal, Fernando Romero, Angel Gil

**Affiliations:** 1 Institute of Nutrition and Food Technology José Mataix, Biomedical Research Centre, Department of Biochemistry and Molecular Biology II, University of Granada, Granada, Spain; 2 Hero Institute for Infant Nutrition, Hero Spain, Alcantarilla, Murcia, Spain; Institut Pasteur de Lille, France

## Abstract

Probiotic bacteria have been shown to modulate immune responses and could have therapeutic effects in allergic and inflammatory disorders. However, little is known about the signalling pathways that are engaged by probiotics. Dendritic cells (DCs) are antigen-presenting cells that are involved in immunity and tolerance. Monocyte-derived dendritic cells (MoDCs) and murine DCs are different from human gut DCs; therefore, in this study, we used human DCs generated from CD34+ progenitor cells (hematopoietic stem cells) harvested from umbilical cord blood; those DCs exhibited surface antigens of dendritic Langerhans cells, similar to the lamina propria DCs in the gut. We report that both a novel probiotic strain isolated from faeces of exclusively breast-fed newborn infants, *Lactobacillus paracasei* CNCM I-4034, and its cell-free culture supernatant (CFS) decreased pro-inflammatory cytokines and chemokines in human intestinal DCs challenged with *Salmonella*. Interestingly, the supernatant was as effective as the bacteria in reducing pro-inflammatory cytokine expression. In contrast, the bacterium was a potent inducer of TGF-β2 secretion, whereas the supernatant increased the secretion of TGF-β1 in response to *Salmonella*. We also showed that both the bacteria and its supernatant enhanced innate immunity through the activation of Toll-like receptor (TLR) signalling. These treatments strongly induced the transcription of the *TLR9* gene. In addition, upregulation of the *CASP8* and *TOLLIP* genes was observed. This work demonstrates that *L. paracasei* CNCM I-4034 enhanced innate immune responses, as evidenced by the activation of TLR signalling and the downregulation of a broad array of pro-inflammatory cytokines. The use of supernatants like the one described in this paper could be an effective and safe alternative to using live bacteria in functional foods.

## Introduction

Probiotics are defined as “live microorganisms that, when administered in adequate amounts, confer a health benefit on the host" [1]. Bifidobacteria and lactic acid bacteria (LAB), primarily lactobacilli, are generally referred to as probiotics because of their health-promoting properties, such as the exclusion or inhibition of pathogens in the gut, the enhancement or maintenance of barrier function and the local and systemic modulation of the host immune system [2], [3]. The clinical applications of lactobacilli and bifidobacteria include preventing and treating allergic diseases, particularly in relieving the symptoms of atopic eczema [4] and allergic rhinitis [5], reducing diarrhoea in children [6], preventing inflammatory bowel disease and viral infection and as adjuvants in vaccines [7]. Despite growing evidence of the immunomodulatory effects of probiotics, there is little information available regarding their mechanisms of action.

Dendritic cells (DCs) are professional antigen-presenting cells and are essential mediators of immunity and tolerance [8], [9]. The control of the immune response by DCs is particularly important in the gut, in which the immune system exists in intimate association with commensal bacteria, such as LAB. In their immature state, DCs reside in peripheral tissues, continuously sampling the microenvironment, sensing the presence of pathogens and releasing chemokines and cytokines to amplify the immune response [10]. Furthermore, DCs interact directly with bacteria that have gained access via M cells [11]. Innate pattern-recognition receptors (PRRs), such as Toll-like receptors (TLRs), NOD-like receptors (NLRs) and C-type lectin receptors (CLRs), play crucial roles in the host recognition of probiotics and other microorganisms [12]. The binding of microbe-associated molecules to these receptors can activate antigen-presenting cells and modulate the activation of important transmission factors, such as nuclear factor kappa B (NFκB) and the production of different cytokines [13]–[15]. Therefore, this recognition provides a platform for modulation of the local innate and systemic adaptive immune response in the host [16]–[19]. Immune assays have shown that the *in vitro* immune response to probiotics is both species- and strain-specific [12]. Interestingly, some probiotics secrete antimicrobial factors that affect both virulence gene expression in pathogenic bacteria [2], [20] and gene expression in the host epithelium [21].

In a previous study, a novel LAB strain was isolated from the faeces of exclusively breast-fed newborn infants and selected based on its probiotic properties, such as adhesion to intestinal mucus, sensitivity to antibiotics and resistance to gastrointestinal juices, biliary salts, NaCl and low pH. We identified this strain as *Lactobacillus paracasei* CNCM I-4034 [22].

The aim of the present study was to investigate the capacity of *L. paracasei* CNCM I-4034 and its cell-free culture supernatant (CFS) to activate human intestinal DCs, to determine how they respond to pathogenic bacteria, specifically *Salmonella typhi,* and to elucidate the molecular mechanisms involved in these interactions. The expression of genes involved in TLR signalling and cytokine secretion was analysed.

## Results

### DCs Co-cultured with the Probiotic and the Enteropathogen Show a Markedly Reduced Pro-inflammatory Response

**Figure 1:**
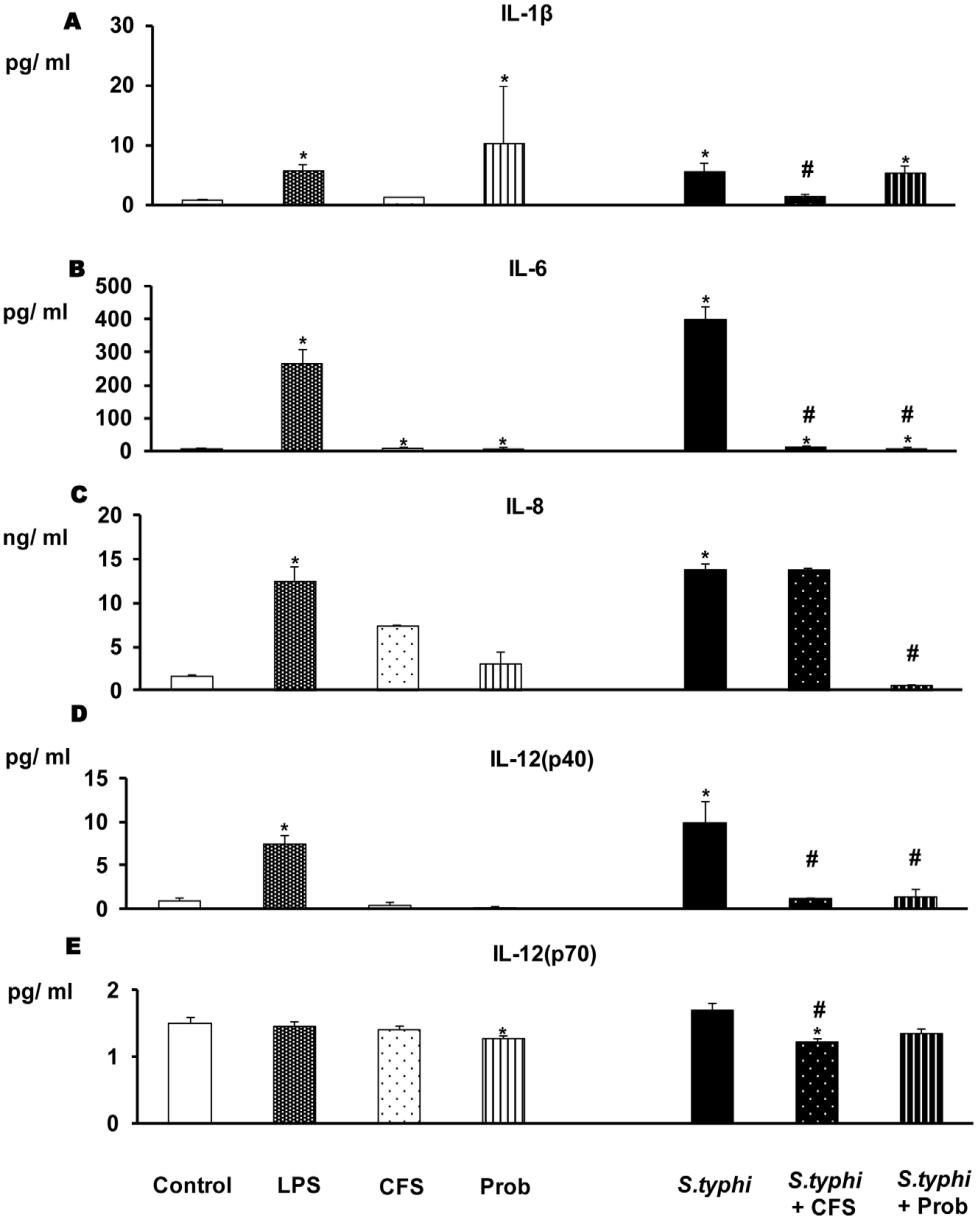
Pro-inflammatory cytokine production in DCs after exposure to *L. paracasei*, *Salmonella* or both. Dendritic cells (DCs) were incubated with the probiotic *L. paracasei* CNCM I-4034 (Prob) or its cell-free supernatant (CFS), *Salmonella* (*S.typhi*), or both. *E. coli* lipopolysaccharide (LPS, 20 ng/ml) was used as a positive control. Negative-control cultures contained unstimulated DCs. IL-1β, IL-6, IL-8, IL-12(p40) and IL-12(p70) production was measured. The data shown are the mean values and SEM of three independent experiments. *p<0.05 compared to controls; #p<0.05 compared to *S.typhi.*

**Figure 2:**
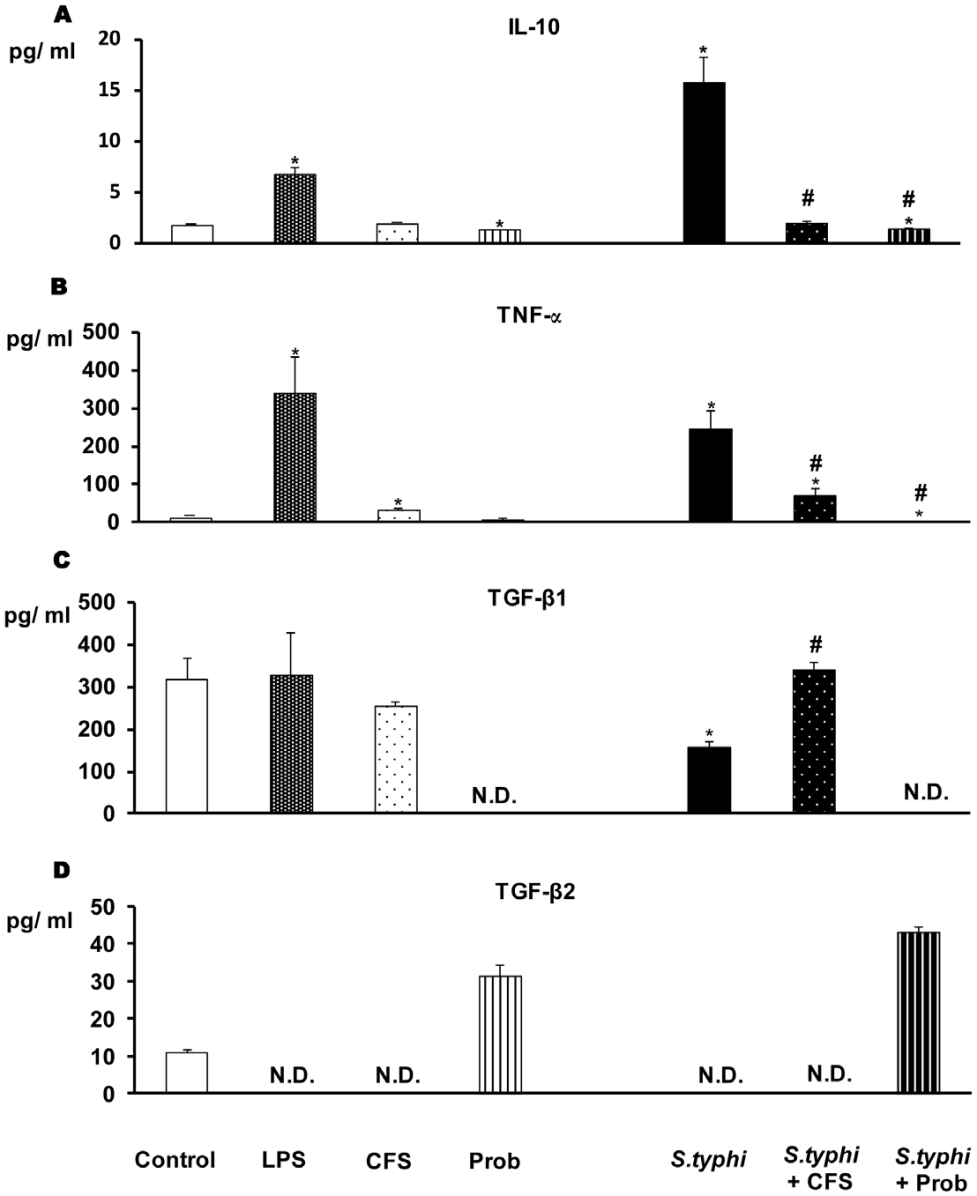
Measure of anti-inflammatory cytokines and TNF-α in DCs after exposure to *L. paracasei*, *Salmonella* or both. DCs were incubated with the probiotic *L. paracasei* CNCM I-4034 (Prob) or its cell-free supernatant (CFS), *Salmonella* (*S.typhi*), or both. IL-10, TNF-α, TGF-β1 and TGF-β2 production was measured. LPS, 20 ng/ml, was used as a positive control. Negative-control cultures contained unstimulated DCs. The data shown are the mean values and SEM of three independent experiments. *p<0.05 compared to controls; #p<0.05 compared to *S.typhi*; N.D indicate no detected.

**Figure 3:**
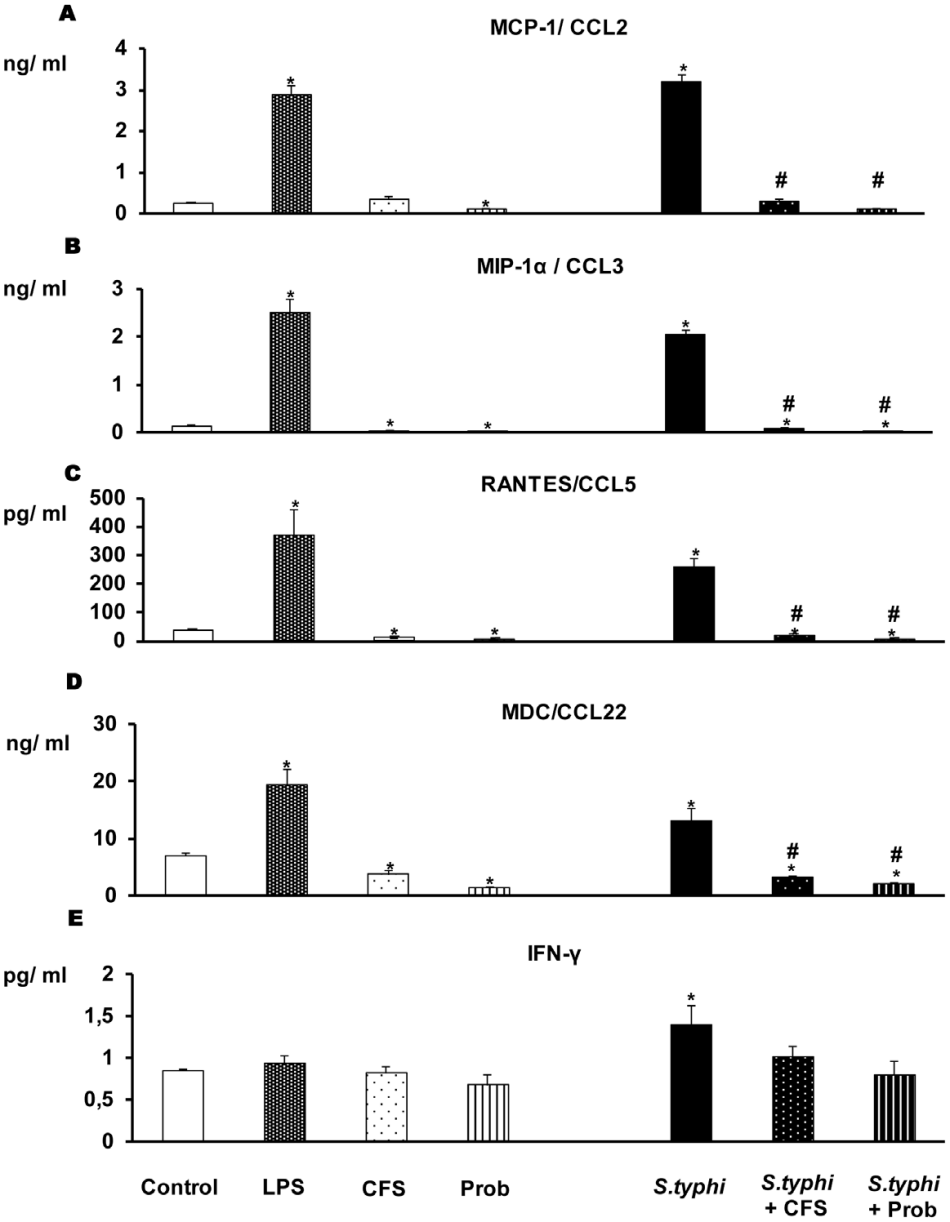
Measure of chemokines and IFNγ in DCs after exposure to *L. paracasei*, *Salmonella* or both. DCs were incubated with the probiotic *L. paracasei* CNCM I-4034 (Prob) or its cell-free supernatant (CFS), *Salmonella* (*S.typhi*), or both. The production of IFNγ and the chemokines MCP-1/CCL2, MIP-1α/CCL3, RANTES/CCL5 and MDC/CCL22 was measured. LPS, 20 ng/ml, was used as a positive control. Negative-control cultures contained unstimulated DCs. The data shown are the mean values and SEM of three independent experiments. *p<0.05 compared to controls; #p<0.05 compared to *S.typhi.*

The immunomodulatory effects of *L. paracasei* CNCM I-4034 were studied in human DCs. The DCs were incubated with the probiotic (live bacteria or CFS) and the pathogen, either individually or together. Our data indicate that probiotic bacteria and their CFS can induce cytokine secretion; this induction was similar in response to probiotic or CFS stimulation. As shown in Figures 1 and 2, the addition of pathogenic bacteria (*S. typhi* CECT 725) or LPS to DCs markedly increased the secretion of pro-inflammatory cytokines. In response to stimulation with the probiotic or its supernatant and the enteropathogen, the secretion of all of the pro-inflammatory cytokines (Figures 1, 2) such as IL-6 and TNF-α and chemokines (Figure 3) including MCP-1/CCL2 and RANTES/CCL5 was drastically reduced.

TGF-β levels clearly showed that *L. paracasei* is a potent inducer of TGF-β. As shown in figure 3, this bacteria increased TGF- β2 production, while the probiotic supernatant increased the secretion of TGF-β1 in response to *Salmonella*. We did not detect the production of TGF-β3 under any of the treatment tested conditions (data not shown).

Interestingly, the supernatant of *L. paracasei* was as effective as the live probiotic bacteria in reducing the secretion of pro-inflammatory cytokines, suggesting that this probiotic secretes metabolites or factors with anti-inflammatory properties.

### The Probiotic Strain *L. paracasei* CNCM I-4034 Induces the Expression of TLR Signalling Pathway Genes in Human DCs

We also evaluated the expression patterns of genes within the TLR signalling pathway. These receptors regulate a variety of different genes, including those that function as transcriptional activators, such as NFκB, whose induction results in the expression of a wide variety of cytokines [23].

**Figure 4:**
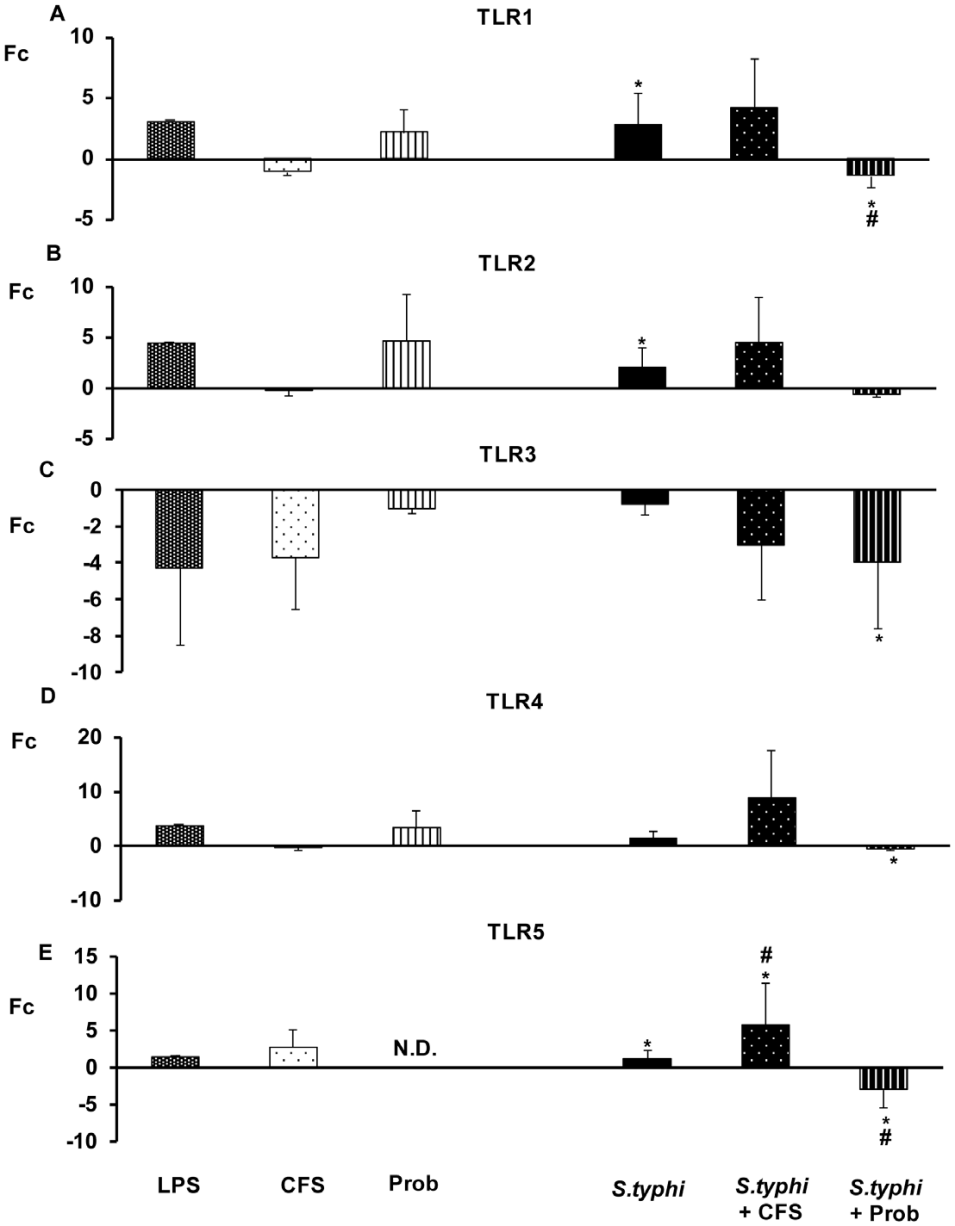
Expression of *TLRs genes* in DCs in the presence of *L. paracasei*, *Salmonella* or both. Comparison of the expression of *TLR1, TLR2, TLR3, TLR4* and *TLR5* in DCs in the presence of the probiotic (Prob), its supernatant (CFS), *Salmonella* (*S.typhi*) or a combination. LPS, 20 ng/ml, was used as a positive control. The data shown are the mean values and SEM of three independent experiments. The fold change (Fc) represents the ratio of the expression in treated DCs to the expression in control cells. *p<0.05 compared to controls; #p<0.05 compared to *S.typhi.* N.D indicate no detected.

**Figure 5:**
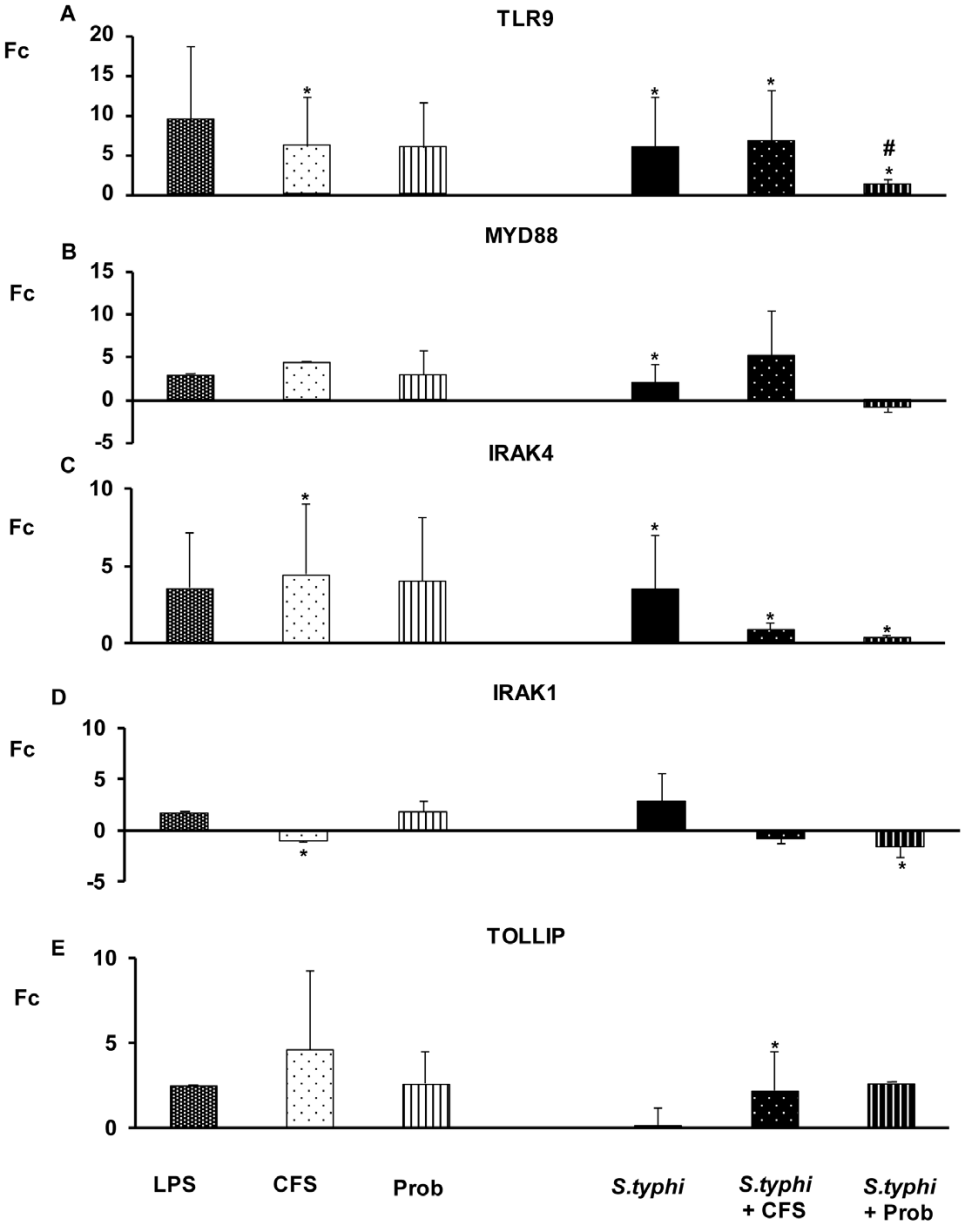
Expression levels of TLR signalling pathway in DCs treated with *L. paracasei*, *Salmonella* or both. Comparison of the expression of *TLR9, MYD88, IRAK-1, IRAK-4* and *TOLLIP* in DCs in the presence of the probiotic (Prob), its supernatant (CFS), *Salmonella* (*S.typhi*) or a combination. LPS, 20 ng/ml, was used as a positive control. The fold change (Fc) represents the ratio of the expression in treated DCs to the expression in control cells. The data shown are the mean values and SEM of three independent experiments. *p<0.05 compared to controls; #p<0.05 compared to *S.typhi.*

**Figure 6:**
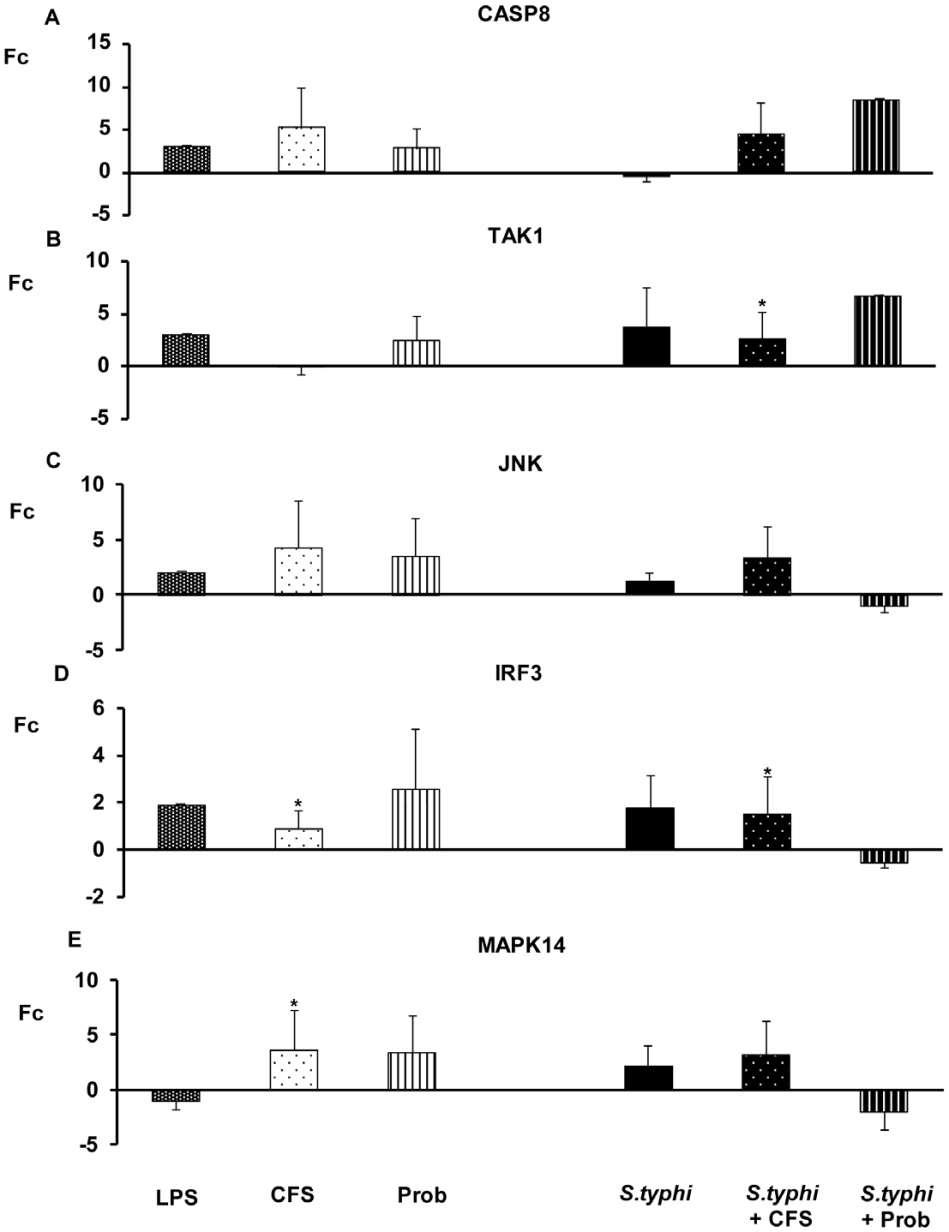
Expression levels of TLR signalling pathway in DCs treated with *L. paracasei*, *Salmonella* or both. Comparison of the expression of *CASP8, TAK-1, JNK, IRF-3* and *MAPK14* in DCs in the presence of the probiotic (Prob), its supernatant (CFS), *Salmonella* (*S.typhi*) or a combination. LPS, 20 ng/ml, was used as a positive control. The fold change (Fc) represents the ratio of the expression in treated DCs to the expression in control cells. The data shown are the mean values and SEM of three independent experiments. *p<0.05 compared to controls; #p<0.05 compared to *S.typhi.*

**Figure 7:**
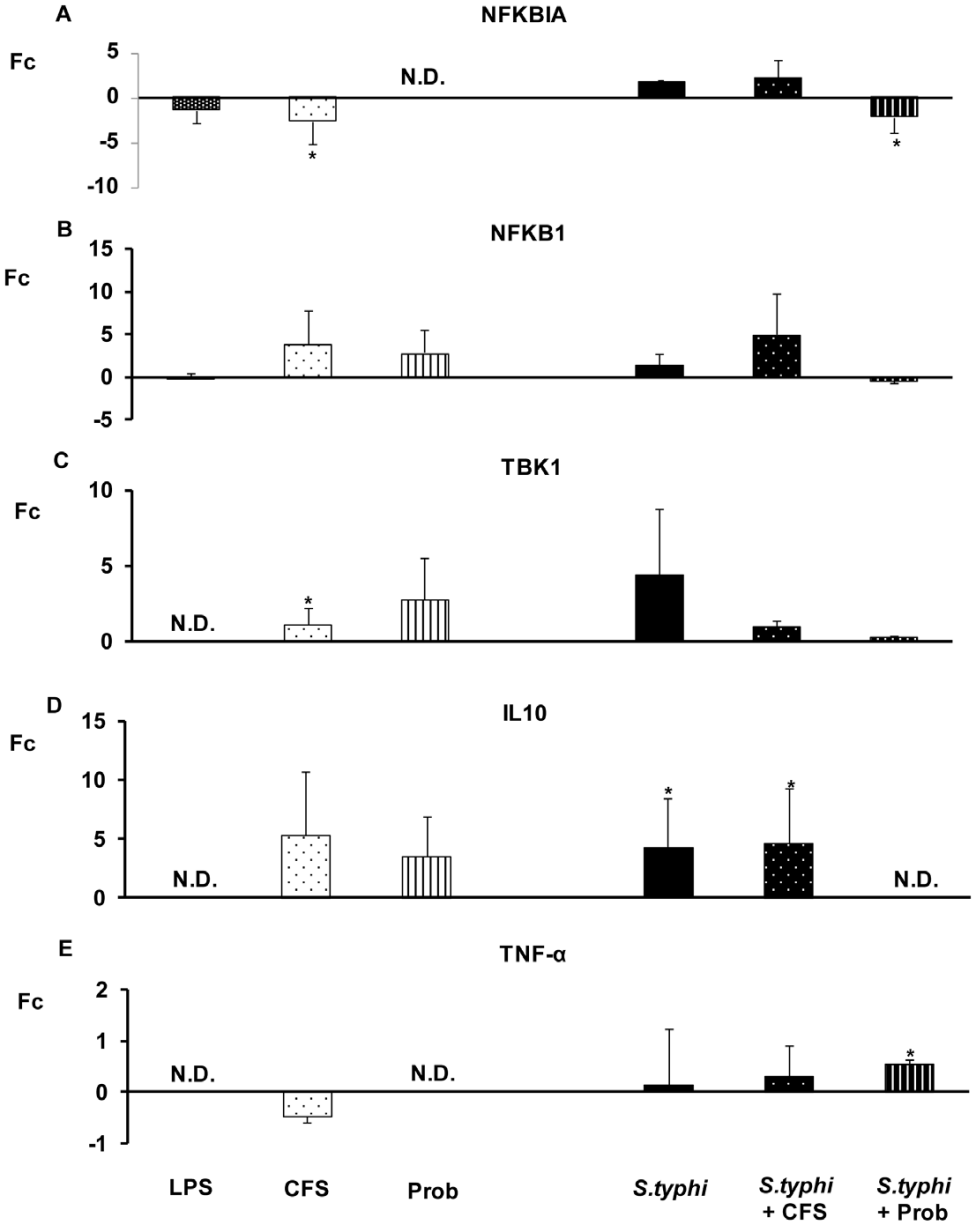
Expression levels of TLR signalling pathway in DCs treated with *L. paracasei*, *Salmonella* or both. Comparison of the expression of *NFKBIA, NFKB-1, TBK-1, IL-10* and *TNF-*α in DCs in the presence of the probiotic (Prob), its supernatant (CFS), *Salmonella* (*S.typhi*) or a combination. LPS, 20 ng/ml, was used as a positive control. The fold change (Fc) represents the ratio of the expression in treated DCs to the expression in control cells. The data shown are the mean values and SEM of three independent experiments. *p<0.05 compared to controls; #p<0.05 compared to *S.typhi.* N.D indicate no detected.

As shown in Figures 4, 5, 6, 7, the exposure of DCs to *S. typhi* for 4 hours resulted in an upregulation of *TLR9* (Figure 5), which was accompanied by an increase in the expression of *TLR1, TLR2, TLR4, TLR5, IRAK4, TAK1, JNK* (Figures 4, 5, 6) and *IL-10* (Figure 7). In addition, it was found that *Salmonella* downregulated *CASP8* (Figure 6) and *TNF-α* (Figure 7) gene expression.

The probiotic bacterium *L. paracasei* CNCM I-4034 and its CFS exhibited similar abilities to regulate TLR pathways (Figures 4, 5, 6, 7). They both induced strong and sustained *TLR9* transcription (Figure 5). Regarding TLR, the probiotic strain alone activated *TLR1*, *TLR2* and *TLR4*, whereas its supernatant increased *TLR5* expression (Figure 4). Interestingly, in response to stimulation with CFS and *Salmonella*, *TLR1*, *TLR2*, *TLR3*, *TLR4*, *TLR5* and *TLR9* expression was increased, while the exposure of DCs with the probiotic and *Salmonella* downregulated *TLR1*–*TLR5* gene expression (Figure 4, 5).

In response to stimulation with the probiotic or its CFS and *Salmonella*, *TOLLIP* (Figure 5), *CASP8* and *TAK1* (Figure 6) and *TNF-α* expression was increased (Figure 7).

## Discussion

There is growing evidence that probiotics, especially lactobacilli and bifidobacteria, have immunomodulatory properties [24]. DCs provide an interface between the innate and adaptive immune systems, acting as professional antigen-presenting cells [8]. Innate PRRs, such as TLRs, are found on epithelial and immune cell surfaces [25] and play a crucial role in the host recognition of bacteria, such as probiotics [10]. During inflammation, these receptors interact with microbe-associated molecules, such as LPS and bacterial DNA, resulting in DC activation. These pathways affect various DC functions, including cytokine production, and the cytokine profile determines the type of T-cell response that will develop [26]. Therefore, this study mainly focused on comparing the expression patterns of genes involved in the TLR signalling pathway and the cytokine profile mediated by exposure to a probiotic or its CFS in the presence or absence of *S. typhi*. We employed an *in vitro* culture system to study the immunological effects and anti-inflammatory properties of the novel probiotic strain *L. paracasei* CNCM I-4034, which was isolated from the faeces of breast-fed newborns.

Specific probiotic strains have been shown to interact with DCs and induce strain-specific effects [27]. In this context, *L. paracasei* CNCM I-4034 and its supernatant dramatically reduced the production of all of the evaluated pro-inflammatory cytokines and chemokines in the presence of *S. typhi*.

The co-incubation of human DCs with *Salmonella* and *L. paracasei* (live bacteria) significantly reduced the ability of *Salmonella* to induce IL-6, IL-8, IL-12p 70 and TNF-α. Interestingly, *L. paracasei* was not able to induce IL-6 secretion. This effect was observed in the presence or absence of pathogens. This observation contrasts with that of Weiss *et al.* who reported that lactobacilli and bifidobacteria are potent inducers of IL-6 [28]. However, this author reported these effects in murine DCs cells and as we mentioned above murine DCs are quite different from human gut DCs in several respects. In this paper, we used human DCs differentiated from umbilical cord blood CD34+ progenitor cells (hematopoietic stem cells). These human DCs are equivalent to those DCs found in Langerhans islets, which extend dendrites and sample antigens, akin to lamina propria DCs in the gut. In addition, as mentioned, *L. paracasei* decreased TNF-α production in response to *Salmonella*. During the inflammatory process, TNF-α functions at the apex of the inflammatory cascade [29] as a consequence of NFκB activation; NFκB regulates the transcriptional activation of a number of genes involved in immune and pro-inflammatory responses [30]. Therefore, this probiotic strongly inhibited the inflammatory response of human DCs to *Salmonella*. In addition, Mileti *et al.* demonstrated that *Lactobacillus* species can be classified as either immunostimulatory or immunomodulatory. Consistent with this work, *L. paracasei* CNCM I-4034 and its supernatant appear to be immunomodulatory and could be used to dampen inflammatory responses [31].

Interestingly, the culture supernatant by itself could decrease *Salmonella*-induced inflammation. Indeed, these data suggest that *L. paracasei* CNCM I-4034 releases some factors of unknown nature, most likely bacteriocins, because the supernatants did not produce large changes in the profiles of the cytokines released by DCs in the absence of the enteropathogen. Accordingly, it is also important to note that this supernatant is concentrated tenfold, as is any other substance present in the growth medium. We suggest that it is unlikely that this inhibition is caused by growth-derived acidic compounds, such as lactic or acetic acid, because the supernatant used was neutralised to a pH of 7.0. Therefore, the nature of the soluble mediator released by *L. paracasei* CNCM I-4034 remains to be determined.

It is clear that the exposure of DCs to pathogen and probiotic resulted in the release of TGF-β, an anti-inflammatory cytokine. *L. paracasei* CNCM I-4034 promoted the stimulation of TGF-β2, whereas the supernatant of the probiotic increased the secretion of TGF-β1. TGF-β1 plays important roles in preventing diseases and controlling intestinal homeostasis [32]. Therefore, the increased secretion of TGF-β1 by DCs stimulated with probiotics may be one mechanism through which probiotics exert anti-inflammatory effects and contribute to immune tolerance. In fact, it has been suggested that this growth factor is crucial for the maturation of tolerogenic Th3 cells [33], [34].

Gene array analysis is a new approach for evaluating the effects of probiotics on immune cells, and it has provided an overall view of the changes in gene expression patterns in probiotic-treated human DCs [35]. Our results showed that *L. paracasei* CNCM I-4034 and its CFS stimulated *TLR9* expression in the presence or absence of enterobacteria. Similarly, several studies have described increased expression of *TLR9* upon the administration of probiotics, such as *L. johnsonii* and *L. casei* [36], [37]. Recently, it has been proposed that apical TLR9 stimulation in intestinal epithelial cells leads to a heightened state of immune surveillance [37]. These results indicate that this TLR is crucial for the regulation of pro-inflammatory cytokines, which is consistent with a recent study that correlated the presence of this TLR with the anti-inflammatory effect induced by probiotics [35]. Moreover, as expected, our probiotic strain activated the expression of *TLR2.* This TLR recognises peptidoglycan, the main component of Gram-positive bacteria, as *Lactobacillus*.

A similar effect has been described by other authors for *L. plantarum* BFE 1685 and *L. rhamnosus* GG, which increase the expression of TLR2 in human epithelial cells [38] and *L. casei* CRL 431 in mice [36]. Recently, it has been reported that this TLR plays a key role in the stimulation of DCs and macrophages in the presence of bacteria from the genus *Lactobacillus* [39]. In line with several studies, our results suggest that probiotic bacteria increase the expression of *TLR2* and *TLR9*, activating innate immunity [38], [40]–[42]. Furthermore, *TLR2* stimulation also appears to enhance the epithelial barrier, and it has been shown that the activation of this TLR plays an essential role in resistance to bacterial invasion [43].

TLR4 expression was decreased in response of the probiotic and *Salmonella* stimulation. Recently, Villena *et al.* reported that *L.jensenii* TL2937 attenuates the inflammatory response triggered by the activation of this TLR in porcine intestinal epithelial cells [44]. In line with several authors, our results revealed that the inhibition of TLR4 pathway blocked IL-8 production [45].

Perhaps most revealing is that the CFS can induce changes in gene expression in the intestine through some factors secreted by the probiotic bacteria. This effect is likely partly responsible for the beneficial effect of probiotics, as highlighted by other authors [40], [46]–[49]. Our results also coincide with those of another recent study indicating that live probiotic bacteria affect the intestinal immune response, whereas secreted components exert anti-inflammatory effects in the gastrointestinal tract [50].

In conclusion, probiotic microorganisms can apparently exert immunoregulatory effects through the secretion of various bacterial compounds that are sensed by host PRRs. This work demonstrates that *L. paracasei* CNCM I-4034 enhanced innate immune responses, as evidenced by the activation of TLR signalling and by the decrease in the production of a broad array of pro-inflammatory cytokines. Our data support the use of a supernatant similar to the one described in this paper as an effective and safe alternative instead of live bacteria. Our results contribute to the understanding of how probiotics exert their immunomodulatory effects.

## Materials and Methods

### Preparation of Bacteria and Probiotic Supernatant

The probiotic strain (designated by the Pasteur Institute as *L. paracasei* CNCM I-4034) was isolated from the faeces of breast-fed newborn children. This strain was previously tested for antibiotic tolerance and resistance to gastrointestinal juices, bile salts, NaCl and low pH. The probiotic was grown in Man-Rogosa-Sharpe (MRS) broth medium (Oxoid, Basingstoke, United Kingdom) at 37°C under anaerobic conditions for 18–24 hours.

To obtain the CFS, the probiotic strain was grown anaerobically at 37°C in MRS for 24 hours. The supernatant was obtained by centrifugation at 12,000×*g* for 10 min, neutralised to pH 7.0 with NaOH (1 N) and concentrated tenfold by lyophilization. Supernatants were sterilized by filtering through a 0.22-µm-pore-size filter (Minisart hydrophilic syringe filter; Sartorius Stedim Biotech GmbH, Goettingen, Germany) and storage at −20°C until use. The supernatant was added 7% v/v.

The pathogenic strain was provided by the Spanish Type Culture Collection (CECT; Burjassot, Spain). The pathogen used in the current study was *S. typhi* CECT 725. The culture was propagated aerobically in tryptone soy broth (Panreac Quimica, Barcelona, Spain).

For the experiments, the pathogen was cultured for 8 hours at 37°C in tryptone soy broth and then subcultured 1∶500 in RPMI 1640 medium (Sigma-Aldrich, St. Louis, MO) containing 10% foetal bovine serum (FBS; Gibco Invitrogen, Paisley, United Kingdom) at 37°C overnight.

### Cell Preparation

DCs generated from umbilical cord blood CD34^+^ progenitor cells (haematopoietic stem cells) were supplied by the MatTek Corporation (Ashland, MA). These cells were seeded in 24-well plates in DC maintenance medium (DC-MM; MatTek) containing cytokines and antibiotics.

### Bacterial Co-culture and DC Stimulation

Cell cultures were seeded in 24-well plates at a final concentration of 2×10^5^ DCs/well. For incubations, DC-MM was replaced with RPMI-1640 media. DCs were directly challenged by the addition of the probiotic live bacteria (10^7^ CFU/ml) or CFS, the pathogen (10^6^ CFU/ml) or both. *Escherichia coli* lipopolysaccharide (LPS; Sigma-Aldrich) at 20 ng/ml was used as a positive control. Negative-control cultures contained unstimulated DCs.

All of the plates were incubated at 37°C in a 5% CO_2_/95% air atmosphere for 4 hours. After incubation, the medium was removed and replaced with fresh DC-MM containing cytokines and antibiotics. After 20 hours, all of the plates were centrifuged at 200×*g*, and the culture supernatants were collected for cytokine analysis. The cells were collected for RNA extraction. The results shown are the mean ± SEM of three independent experiments.

### Cytokine and Chemokine Quantification in Culture Supernatants

IL-1β, IL-6, IL-8, IL-10, IL-12(p40), IL-12(p70), TNF-α, IFNγ, MCP-1/CCL2, MIP-1α/CCL3, RANTES/CCL5, MDC/CCL22 and TGF-β were measured with MILLIplex™ immunoassays (Linco Research Inc., MO) using the Luminex 200 system according to the manufacturer’s instructions. The results shown are the mean ± SEM of three independent experiments.

### Reverse Transcriptase (RT) Reaction and Polymerase Chain Reaction (PCR)

As previously described, DCs were stimulated with bacteria for 4 hours and collected 20 hours later. The DCs were lysed and total RNA was extracted using the RNAqueous Kit (Ambion, Paisley, United Kingdom) and treated with Turbo DNase (Ambion) according to the manufacturer’s recommendations. The RNA quality was verified with a Model 2100 Bioanalyser (Agilent, Santa Clara, USA) and the RNA concentration was determined using a RediPlate 96 RiboGreen RNA Quantitation Kit (Gibco, Invitrogen). Real-time RT-PCR analysis of the samples was performed using a Human TLR Signaling Pathway PCR Array (SABiosciences Corporation, Frederick, MD), which includes primer pairs specific for 20 genes involved in TLR-mediated signalling pathways: *TLR1, TLR2, TLR3, TLR4, TLR5, TLR9, MYD88, TNF, IRAK-1, IRAK-4, TOLLIP, CASP8, IL-10, TAK-1, JNK, NFKBIA, NFKB-1, TBK-1, MAPK14* and *IRF-3*. The housekeeping gene *GAPDH* was used as a control.

Briefly, cDNA was synthesised from total RNA with an RT^2^ First-Strand Kit (SABiosciences). The cDNA was then subjected to real-time PCR with an RT^2^ Real-time PCR SYBR Green/ROX Kit (SABiosciences) on an ABI Prism 7500 sequence detector (Applied Biosystems, Foster City, CA). The PCR conditions were 1 cycle of 95°C for 10 min followed by 40 cycles of 95°C for 15 s and 60°C for 1 min. The expression levels of the target genes were normalised to those of untreated DCs (control).

The expression level of each gene was analysed with RT^2^ Profiler PCR Array Data Analysis software (version 3.4; SABiosciences). The changes in expression or activity levels were expressed as fold changes (Fc). These results reflect the fold increase relative to the control samples (untreated DCs).

### Statistical Analysis

NCSS 2007 software (Kaysville, UT) was used for the statistical analysis. The differences in cytokine expression levels and gene expression between treatments were evaluated with the Mann-Whitney *U*-test. The results are presented as the mean + SEM of three independent experiments. All P values<0.05 were considered to be significant and are indicated in the text with an asterisk compared to the controls. In addition, differences between DCs treated with *Salmonella* and *Salmonella* and the probiotic/CFS were also evaluated. P values<0.05 were considered to be significant and are indicated with a pound sign (#).
